# Impact of previous percutaneous coronary intervention on cardiovascular outcomes and mortality after lung cancer surgery: A nationwide study in Korea

**DOI:** 10.1111/1759-7714.13563

**Published:** 2020-07-12

**Authors:** Dong Woog Yoon, Dong Wook Shin, Jong Ho Cho, Jong‐Hwan Lee, Jeong Hoon Yang, Kyungdo Han, Sang Hyun Park

**Affiliations:** ^1^ Department of Thoracic and Cardiovascular Surgery Armed Forces Capital Hospital Seongnam South Korea; ^2^ Supportive Care Center/Department of Family Medicine, Samsung Medical Center Sungkyunkwan University School of Medicine Seoul South Korea; ^3^ Department of Digital Health, SAIHST Sungkyunkwan University Seoul South Korea; ^4^ Department of Thoracic and Cardiovascular Surgery, Samsung Medical Center Sungkyunkwan University School of Medicine Seoul South Korea; ^5^ Department of Anesthesiology and Pain Medicine, Samsung Medical Center Sungkyunkwan University School of Medicine Seoul South Korea; ^6^ Division of Cardiology, Department of Medicine, Samsung Medical Center Sungkyunkwan University School of Medicine Seoul South Korea; ^7^ Department of Medical Statistics The Catholic University of Korea Seoul South Korea

**Keywords:** Cardiovascular disease, lung cancer, percutaneous coronary intervention, survival

## Abstract

**Background:**

The number of patients with operable lung cancer with a history of percutaneous coronary intervention (PCI) has increased. However, cardiovascular outcomes and mortality, according to the time from PCI to surgery, and the follow‐up time after surgery are largely unknown. Here, we aimed to compare the cardiovascular outcomes and mortality of these patients with a history of PCI to those of patients without a history of PCI.

**Methods:**

Using the Korean National Health Insurance Service Database, we selected 30 750 patients who underwent surgery for lung cancer between 2006 and 2014. Study outcome variables were all‐cause mortality, revascularization, intensive care unit (ICU) readmission, and stroke incidence. Patients were followed‐up until 2016.

**Results:**

Of the 30 750 patients, 513 (1.7%) underwent PCI before surgery. The PCI group did not show an increased risk of death, ICU readmission, or stroke within one year of surgery, despite an increased risk of revascularization. However, one year after surgery, they showed a higher risk of death and revascularization than the non‐PCI group. The risk of revascularization was highest when the interval between PCI and surgery was <1 year and remained high when the interval was >3 years.

**Conclusions:**

Patients who underwent PCI before surgery for lung cancer were at a higher risk of death than those in the non‐PCI group at one year after surgery. In addition, they showed higher short‐ and long‐term risks of revascularization than patients in the non‐PCI group. Careful long‐term management of cardiovascular risk is necessary for this population.

## Graphical abstract

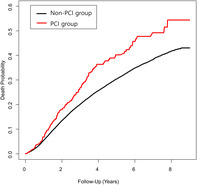

The PCI group had higher short‐ and long‐term risks of revascularization. Careful long‐term management of cardiovascular risk is required for the PCI group.

## Introduction

As percutaneous coronary intervention (PCI) has become the primary therapy for symptomatic coronary disease in the last decade,^1^ the number of patients who have undergone PCI before resection for non‐small cell lung cancer (NSCLC) has increased. Recent data have indicated that coronary artery disease is present in 7%–16% of patients affected by operable lung cancer.^2^, ^3^ Therefore, cardiovascular outcomes and mortality in operable lung cancer patients with prior PCI have been a major concern for thoracic surgeons.

Prior PCI can cause several problems during the perioperative period in patients undergoing lung cancer surgery. For example, discontinuation of antiplatelet therapy in noncardiac surgery is associated with a high risk of major adverse cardiac events (MACE) following pulmonary resection for lung cancer. In addition, resuming antiplatelet therapy after surgery may increase the risk of bleeding complications. There are few reports on the degree of cardiovascular risk and on whether risk can be limited by careful perioperative management. Cardiovascular risk and mortality may differ according to the time interval between PCI and surgery, because the requirement of dual antiplatelet therapy (DAPT) and probability of late stent thrombosis also differ over time.^4^, ^5^ Although a previous study examined risk in lung cancer patients who underwent PCI within one year prior to surgery, the effect of the time interval between PCI and surgery was not investigated. That study included patients undergoing surgery between 1998 and 2005, during which time clinical practices for PCI and postoperative care were different from current practices.^6^

The influence of cardiovascular comorbidity on survival in patients with operable lung cancer is controversial. Some studies have shown an increased risk of death,^7^, ^8^ while others have not.^9^, ^10^, ^11^ Although cardiovascular disease is well known to become an increasingly important cause of death over the course of follow‐up,^12^, ^13^ the mechanism by which prior PCI affects short‐term and long‐term outcomes after lung cancer surgery has not been reported.

Therefore, this population‐based study used a national database to investigate the impact of prior PCI on cardiovascular outcomes (revascularization, stroke, and intensive care unit [ICU] readmission) and mortality in patients undergoing pulmonary resection for NSCLC. We also examined cardiovascular outcomes and mortality according to the postoperative period and interval between PCI and surgery.

## Methods

### Data source: Korean National Health Insurance Service

The Korean National Health Insurance Service (NHIS) is a single government payer providing universal public health insurance to approximately 97% of the Korean population. Individuals in the lowest income bracket (approximately 3% of the population) are covered by Medical Aid funded by government taxes, but the NHIS is also responsible for case management and reimbursement.

Medical service providers are mostly private and are mainly reimbursed on a fee‐for‐service basis. Therefore, the NHIS collects all data necessary for reimbursement, including disease codes (based on the International Classification of Diseases [ICD]‐10), information on outpatient clinic visits and hospitalizations, a detailed list of diagnostic tests, procedures, and other medical treatments, drug prescriptions and costs incurred, as well as demographic data of the enrollees, such as age, sex, residential area, and income status (in Korea, the insurance premium level is determined by the income level and not by health risk).

The NHIS also provides a free biennial health screening program for all national health insurance members over 40 years of age and those who are employed, regardless of their age.^14^ This program aims to detect cardiovascular risk factors including hypertension, diabetes, and dyslipidemia for subsequent educational counseling or treatment referral. The program includes questionnaires about medical history and health behaviors, anthropometric measurements, and laboratory tests, and the NHIS database contains the results of these screening tests.

### Study population

**Figure 1:**
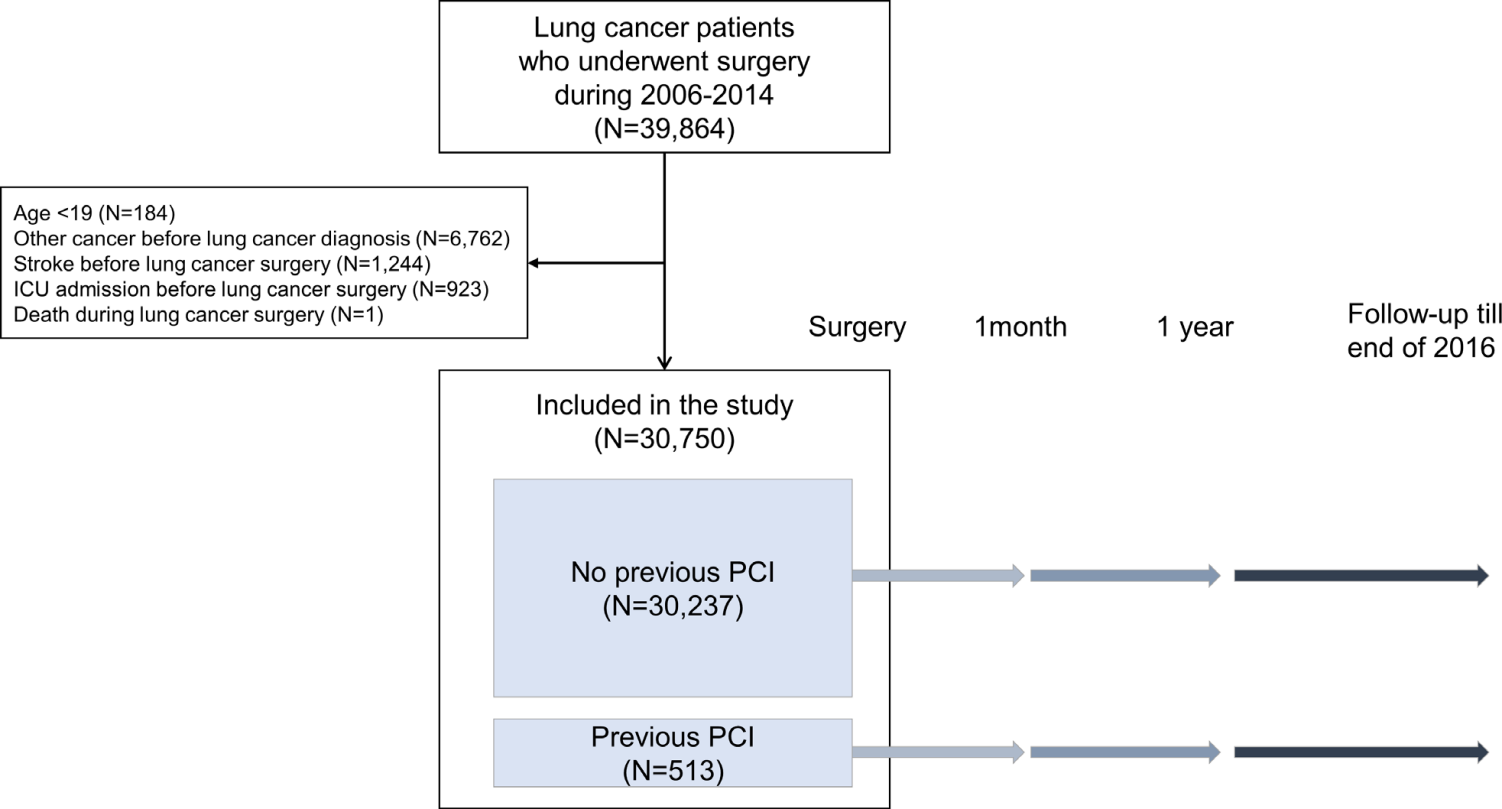
Study participants.

We selected a total of 39 864 patients who underwent surgery for lung cancer (ICD‐10 code C34) between 1 January 2006 and 31 December 2014. We excluded patients who: (i) were younger than 19 years (*N* = 184); (ii) had been diagnosed with another cancer (C00‐C97 except C34, *N* = 6762) before being diagnosed with lung cancer; (iii) experienced stroke (ICD code I60‐I69) before lung cancer surgery (*N* = 1244); (iv) experienced ICU readmission before lung cancer surgery (*N* = 923); and (v) died during lung cancer surgery (*N* = 1). Finally, 30 750 lung cancer patients were included in the analyses. Among these patients, 513 underwent PCI before lung cancer surgery (PCI group). The control group was composed of 30 237 patients who did not undergo PCI before surgery (non‐PCI group; Fig 1). In addition, 19 858 patients who participated in the national health screening program and thus had information on smoking status and body mass index (BMI) comprised the screening subset population (screening subgroup). The Institutional Review Board (IRB) of the Samsung Medical Center approved this study (IRB number: SMC 2018–06‐031).

**Figure 2:**
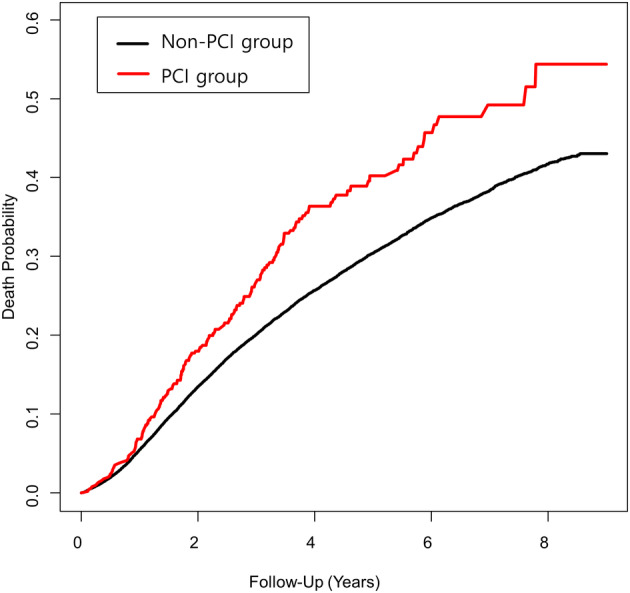
Kaplan‐Meier overall survival curve in surgically treated lung cancer patients by prior PCI status (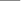) Non‐PCI group, (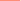) PCI group.

### Independent variable: PCI before lung cancer surgery

PCI before surgery was defined as having a procedural code claim for percutaneous transcatheter placement of an intracoronary stent or percutaneous transluminal coronary atherectomy before the date when lung cancer surgery was performed. To examine the potential differential impact, the timing of PCI before surgery was classified as <1 year, 1–3 years, or > 3 years, based on the minimum DAPT duration of 12 months recommended by clinical practice guidelines^4^ and duration of 30 months suggested by other groups favoring longer DAPT.^15^

### Outcome variables

We selected four study outcome variables based on the literature and clinical insights: (i) all‐cause mortality,^16^, ^17^, ^18^, ^19^, ^20^ (ii) ICU readmission,^16^, ^21^ (iii) revascularization after surgery,^16^, ^17^, ^20^ and (iv) incidence of stroke.^18^, ^22^ Mortality data were obtained from the National Statistical Office. ICU admission on the day of lung cancer surgery was not considered readmission because most patients undergoing major lung resection were admitted to the ICU immediately after surgery. Coronary revascularization was defined as PCI or coronary artery bypass grafting after the date when lung cancer surgery was performed. Stroke incidence was defined as ischemic stroke diagnosis (ICD code I60‐I69) recorded after the day of lung cancer surgery. In addition, we analyzed the occurrence of major adverse cardiac events (MACE), which included acute myocardial infarction, revascularization, and acute ischemic stroke, identified from the Korean National Health Insurance Service claims database.^23^ Patients were followed up until 31 December 2016. Median follow‐up times for each outcome were 3.27, 3.09, 3.23, and 3.22 years, respectively. Previous studies have shown that the risk of dying of cancer decreased over time in lung cancer patients, while the risk of dying of cardiovascular disease increased.^12^, ^13^ Based on these studies, we divided the outcome period as <1 year and ≥ 1 year after lung cancer surgery to examine the impact of preoperative PCI on both short‐ and long‐term outcomes.

### Covariates

Income level was categorized into quartiles based on insurance premium brackets. As Medical Aid covers only 3% of the population, these patients were merged into the lowest income quartile group. Place of residence was categorized into three major types: metropolitan, urban, and rural areas.

Comorbidity information was defined as claims and prescription codes for specific diseases within one year prior to lung cancer diagnosis and included hypertension (I10–I13, I15, and prescription for antihypertensive medication), diabetes mellitus (E11–E14 and prescription for antidiabetic medication), ischemic heart disease (I20–I25), stroke (I63, I64), and chronic obstructive pulmonary disease (COPD) (J40–J47), based on the prevalence and risk of complications during surgery and incidence of study outcomes. To account for the risk of additional mortality due to comorbidities, we determined Charlson comorbidity scores^24^ based on claims data for a cancer diagnosis made in the previous year or based on the index date.

Surgeries were categorized into wedge resection, segmentectomy, lobectomy, and pneumonectomy. Chemotherapy and radiotherapy were not further categorized owing to the complexity of the regimens.

For screening of subsets, information on smoking status (current, past, non‐smoker) and BMI (categorized into <18.5, 18.5–23, 23–25, 25–30, and ≥ 30 kg/m^2^) was also available.

### Statistical analysis

Descriptive statistics were used to determine the basic characteristics of the study population according to PCI before surgery. Cox proportional hazards regression analysis was used to determine the relative risk of each study outcome. Multivariate model 1 was adjusted for age, sex, income, place of residence, hypertension, diabetes, dyslipidemia, COPD, surgery type, radiotherapy, and chemotherapy. Multivariate model 2 was applied to the screening subgroup, with further adjustment for BMI and smoking status.

All statistical analyses were performed using SAS version 9.1 (SAS institute, Cary, NC, USA), and *P*‐value <0.05 was considered statistically significant.

## Results

### Study participants

Lung cancer patients who underwent PCI before surgery were older than the control subjects (66.9 vs. 61.9 years) and more likely to be male (82.7% vs. 64.1%). They had more comorbidities, including hypertension, diabetes, dyslipidemia, and COPD, with a higher mean Charlson comorbidity index score (3.1 vs. 1.7). No differences in income level, place of residence, surgery type, and treatment modalities were noted. In the screening subgroup, those who underwent PCI had a higher percentage of current smokers (31.1% vs. 29.0%) and ex‐smokers (34.1% vs. 19.7%), and a higher BMI (Table 1).

**Table 1:** Baseline characteristics of patients according to percutaneous coronary intervention (PCI) status before lung cancer surgery

	PCI before lung cancer surgery		*P*‐value
	No	Yes	
Total N	30 237	513	
Age			
Mean ± SD	61.9 ± 10.0	66.9 ± 7.1	<0.001
20–49	3384 (11.2)	6 (1.2)	<0.001
50–59	8056 (26.6)	77 (15.0)	
60–69	11 493 (38.0)	245 (47.8)	
70–79	6821 (22.6)	164 (32.0)	
80‐	483 (1.6)	21 (4.1)	
Sex			<0.001
Male	19 369 (64.1)	424 (82.7)	
Female	10 868 (35.9)	89 (17.4)	
Charlson comorbidity index			
Mean ± SD	1.7 ± 1.8	3.1 ± 2.2	<0.001
0	9810 (32.4)	52 (10.1)	<0.001
1	7723 (25.5)	84 (16.4)	
2	5077 (17.0)	95 (18.5)	
3	3324 (11.0)	86 (16.8)	
4‐	4303 (14.2)	196 (38.2)	
Comorbidity			
Hypertension	12 607 (41.7)	428 (83.4)	<0.001
Diabetes mellitus	5119 (16.9)	215 (41.9)	<0.001
Dyslipidemia	6982 (23.1)	440 (85.8)	<0.001
COPD	17 042 (56.4)	352 (68.6)	<0.001
Income level			0.399
Lowest quartile + medical aid	7486 (24.8)	123 (24.0)	
Second quartile	5947 (19.7)	88 (17.2)	
Third quartile	7343 (24.3)	128 (25.0)	
Highest quartile	9461 (31.3)	174 (33.9)	
Place of residence			0.949
Metropolitan	18 359 (60.7)	315 (61.4)	
City	8189 (27.1)	137 (26.7)	
Rural	3689 (12.2)	61 (11.9)	
Surgery types			
Wedge	5393 (17.8)	100 (19.5)	0.331
Segmentectomy	943 (3.1)	20 (3.9)	0.315
Lobectomy	24 980 (82.6)	415 (80.9)	0.309
Pneumonectomy	1381 (4.6)	15 (2.9)	0.076
Treatment group			0.058
Surgery only	17 273 (57.1)	315 (61.4)	
Surgery + chemotherapy	8041 (26.6)	125 (24.4)	
Surgery + radiotherapy	1122 (3.7)	24 (4.7)	
Surgery + chemotherapy + Radiotherapy	3801 (12.6)	49 (9.6)	
Past history of heart disease			
Ischemic heart disease	4615 (15.3)	513 (100)	<0.001
Myocardial infarction	303 (1.0)	97 (18.9)	<0.001
Congestive heart failure	1246 (4.1)	84 (16.4)	<0.001
Coronary artery bypass graft	12 (0.0)	2 (0.4)	<0.001
Timing of PCI before surgery			
<1 year	NA	120 (23.4)	
1–3 years	NA	152 (29.6)	
>3 years	NA	241 (47.0)	
Screening subset (*N* = 19 858)	19 562	296	
Smoking			<0.001
Non‐smoker	10 032 (51.3)	103 (34.8)	
Ex‐smoker	3850 (19.7)	101 (34.1)	
Current	5680 (29.0)	92 (31.1)	
Body mass index (BMI), kg/m^2^			0.002
<18.5	650 (3.3)	8 (2.7)	
18.5–23	7633 (39.0)	85 (28.7)	
23–25	5353 (27.4)	87 (29.4)	
25–30	5458 (27.9)	109 (36.8)	
> = 30	468 (2.4)	7 (2.4)	

SD, standard deviation.

### Risk of study outcomes

Prior PCI was associated with increased risk of death (adjusted hazard ratio [aHR] = 1.36; 95% confidence interval [CI]: 1.16–1.60) and revascularization (aHR = 3.76; 95% CI: 2.76–5.11). These effects became more prominent in the screening subgroup, when further adjusted for BMI and smoking (aHR, 1.54; 95% CI: 1.25–1.89 for death and aHR 5.13; 95% CI: 3.47–7.60 for revascularization). However, the risk of ICU readmission (HR = 1.06; 95% CI: 0.80–1.39) and stroke (HR = 1.33; 95% CI: 0.87–2.04) was not significantly increased in the PCI group (Table 2, multivariate model 2).

**Table 2:** Risk of study outcomes by previous PCI

Outcomes	PCI before surgery	N	No. of events	Person ‐years	Rate (per 1000 person ‐years)	Crude model	Multivariate model 1	Multivariate model 2	Multivariate model 3
All patients									
Death	No	30 237	7947	112 286.13	70.77	1(ref.)	1(ref.)	1(ref.)	
	Yes	513	166	1660.51	99.97	1.41 (1.21–1.64)	1.28 (1.09–1.50)	1.36 (1.16–1.60)	
ICU readmission	No	30 237	2655	107 482.06	24.70	1(ref.)	1(ref.)	1(ref.)	
	Yes	513	55	1565.65	35.13	1.36 (1.04–1.77)	1.02 (0.78–1.34)	1.06 (0.80–1.39)	
Revascularization	No	30 237	384	111 375.11	3.45	1(ref.)	1(ref.)	1(ref.)	
	Yes	513	53	1559.25	33.99	9.82 (7.36–13.09)	3.78 (2.78–5.13)	3.76 (2.76–5.11)	
Stroke	No	30 237	781	111 073.57	7.03	1(ref.)	1(ref.)	1(ref.)	
	Yes	513	23	1612.98	14.26	2.01 (1.33–3.04)	1.29 (0.84–1.98)	1.33 (0.87–2.04)	
MACE	No	30 237	1869	108 794.22	17.18	1(ref.)	1(ref.)	1(ref.)	
	Yes	513	115	1423.00	80.82	4.5 (3.73,5.43)	2.43 (1.99,2.96)	2.48 (2.03, 3.02)	
Screening subgroup									
Death	No	19 562	4670	70 839.56	65.92	1(ref.)	1(ref.)	1(ref.)	1(ref.)
	Yes	296	96	919.87	104.36	1.59 (1.30–1.95)	1.45 (1.18–1.79)	1.54 (1.25–1.90)	1.54 (1.25–1.89)
ICU readmission	No	19 562	1597	67 911.73	23.52	1(ref.)	1(ref.)	1(ref.)	1(ref.)
	Yes	296	28	871.57	32.13	1.30 (0.90–1.89)	1.00 (0.68–1.46)	1.02 (0.70–1.50)	1.02 (0.69–1.49)
Revascularization	No	19 562	219	70 323.9	3.11	1(ref.)	1(ref.)	1(ref.)	1(ref.)
	Yes	296	34	853.9	39.82	12.76 (8.88–18.32)	5.11 (3.46–7.55)	5.11 (3.45–7.55)	5.13 (3.47–7.60)
Stroke	No	19 562	464	70 100.28	6.62	1(ref.)	1(ref.)	1(ref.)	1(ref.)
	Yes	296	12	890.06	13.48	2.03 (1.14–3.60)	1.37 (0.76–2.48)	1.39 (0.77–2.51)	1.42 (0.79–2.57)
MACE	No	19 562	1085	68 780.76	15.77	1(ref.)	1(ref.)	1(ref.)	1(ref.)
	Yes	296	68	774.57	87.79	5.31 (4.16–6.79)	3.00 (2.32–3.89)	3.05 (2.35–3.95)	3.10 (2.39–4.01)

MACE, major adverse cardiac events including acute myocardial infarction, revascularization, and acute ischemic stroke.

Multivariate model 1: adjusted for age, sex, income, place of residence, hypertension, diabetes, dyslipidemia, COPD.

Multivariate model 2: adjusted for age, sex, income, place of residence, hypertension, diabetes, dyslipidemia, COPD, surgery type, radiotherapy, chemotherapy.

Multivariate model 3: (screening subgroup only): adjusted for age, sex, income, place of residence, hypertension, diabetes, dyslipidemia, COPD, surgery type, radiotherapy, chemotherapy, BMI, and smoking.

### Study outcomes according to time interval between PCI and surgery

The risks of death and revascularization increased during the three‐year interval between PCI and surgery, and the risks were the highest within the first year (death, HR = 1.65; 95% CI: 1.23–2.20, and revascularization, HR = 6.76; 95% CI: 4.36–10.47). Risk of revascularization was still higher when PCI was performed more than three years prior (aHR 3.21; 95% CI: 2.04–5.05). ICU readmission and stroke were not associated with prior PCI for any interval (Table 3, multivariate model 2). When the group with less than one year between PCI and surgery was additionally divided into less than six months and six months to one year, patients who had PCI less than six months before lung cancer surgery had a higher risk of death (aHR 1.87, 1.33–2.63) and revascularization (aHR 8.72, 95% CI: 5.35–14.21), but no increased risk of stroke (aHR 1.15, 95% CI: 0.37–3.58) (Table S1).

**Table 3:** Risk of study outcomes according to interval between PCI and surgery

Outcomes	PCI before surgery	N	No. of events	Person‐years	Rate (per 1000 person‐years)	Crude model	Multivariate model 1	Multivariate model 2	Multivariate model 3
All patients									
Death	No	30 237	7947	112 286.13	70.78	1(ref.)	1(ref.)	1(ref.)	
	‐ 1 year	120	47	402.03	116.91	1.65 (1.24–2.20)	1.47 (1.1–1.96)	1.65 (1.23–2.20)	
	1–3 years	152	51	513.44	99.33	1.40 (1.06–1.84)	1.30 (0.99–1.72)	1.45 (1.09–1.91)	
	3 years ‐	241	68	745.04	91.27	1.28 (1.01–1.63)	1.16 (0.91–1.47)	1.17 (0.92–1.49)	
ICU readmission	No	30 237	2655	107 482.06	24.70	1(ref.)	1(ref.)	1(ref.)	
	‐ 1 year	120	17	376.05	45.20	1.76 (1.09–2.84)	1.30 (0.80–2.10)	1.4 (0.87–2.27)	
	1–3 years	152	15	495.52	30.27	1.19 (0.72–1.98)	0.93 (0.56–1.55)	0.97 (0.58–1.62)	
	3 years ‐	241	23	694.08	33.14	1.25 (0.83–1.89)	0.93 (0.62–1.41)	0.94 (0.62–1.42)	
Revascularization	No	30 237	384	111 375.11	3.45	1(ref.)	1(ref.)	1(ref.)	
	‐ 1 year	120	23	357.54	64.33	18.55 (12.18–28.27)	6.66 (4.30–10.30)	6.76 (4.36–10.47)	
	1–3 years	152	9	492.95	18.26	5.28 (2.73–10.22)	2.19 (1.12–4.28)	2.18 (1.11–4.25)	
	3 years ‐	241	21	708.77	29.63	8.57 (5.52–13.30)	3.26 (2.07–5.13)	3.21 (2.04–5.05)	
Stroke	No	30 237	781	111 073.57	7.03	1(ref.)	1(ref.)	1(ref.)	
	‐ 1 year	120	3	391.41	7.66	1.08 (0.35–3.37)	0.70 (0.22–2.18)	0.72 (0.23–2.26)	
	1–3 years	152	9	487.75	18.45	2.6 (1.35–5.02)	1.69 (0.87–3.29)	1.78 (0.92–3.47)	
	3 years ‐	241	11	733.82	14.99	2.10(1.16–3.81)	1.35(0.74–2.46)	1.36 (0.75–2.50)	
MACE	No	30 237	1869	108 794.22	17.18	1(ref.)	1(ref.)	1(ref.)	
	‐ 1 year	120	35	329.16	106.33	5.93 (4.25–8.29)	3.12 (2.22–4.39)	3.22 (2.92–4.53)	
	1–3 years	152	35	419.4	83.45	4.66 (3.33–6.51)	2.55 (1.82–3.58)	2.66 (1.89–3.73)	
	3 years ‐	241	45	674.53	66.71	3.71 (2.76–4.99)	2.01 (1.49–2.72)	2.01 (1.49–2.72)	
Screening subgroup									
Death	No	19 562	4670	70 839.56	65.92	1(ref.)	1(ref.)	1(ref.)	1(ref.)
	‐ 1 year	72	25	220.68	113.29	1.72 (1.16–2.55)	1.53 (1.03–2.27)	1.72 (1.15–2.55)	1.73 (1.16–2.57)
	1–3 years	90	33	293.79	112.33	1.71 (1.21–2.40)	1.62 (1.15–2.29)	1.75 (1.24–2.47)	1.75 (1.24–2.48)
	3 years ‐	134	38	405.4	93.73	1.43 (1.04–1.97)	1.29 (0.94–1.78)	1.32 (0.95–1.82)	1.30 (0.94–1.79)
ICU readmission	No	19 562	1597	67 911.73	23.52	1(ref.)	1(ref.)	1(ref.)	1(ref.)
	‐ 1 year	72	7	212.55	32.93	1.32 (0.63–2.78)	0.98 (0.47–2.07)	1.03 (0.49–2.18)	1.03 (0.49–2.17)
	1–3 years	90	8	283.82	28.19	1.17 (0.59–2.35)	0.94 (0.47–1.89)	0.97 (0.48–1.95)	0.97 (0.48–1.96)
	3 years ‐	134	13	375.2	34.65	1.38 (0.80–2.39)	1.05 (0.60–1.82)	1.05 (0.61–1.83)	1.04 (0.60–1.80)
Revascularization	No	19 562	219	70 323.9	3.11	1(ref.)	1(ref.)	1(ref.)	1(ref.)
	‐ 1 year	72	18	190.43	94.52	30.18 (18.64–48.87)	10.98 (6.61–18.23)	11.16 (6.71–18.55)	11.53 (6.93–19.19)
	1–3 years	90	7	279.48	25.05	8.06 (3.80–17.10)	3.52 (1.63–7.57)	3.57 (1.66–7.70)	3.61 (1.68–7.79)
	3 years ‐	134	9	383.98	23.44	7.51 (3.85–14.63)	2.99 (1.51–5.92)	2.92 (1.47–5.80)	2.87 (1.44–5.71)
Stroke	No	19 562	464	70 100.28	6.62	1(ref.)	1(ref.)	1(ref.)	1(ref.)
	‐ 1 year	72	1	215.09	4.65	0.70 (0.10–4.99)	0.48 (0.07–3.40)	0.48 (0.07–3.41)	0.48 (0.07–3.45)
	1–3 years	90	8	269.7	29.66	4.47 (2.22–8.99)	3.14(1.54–6.40)	3.20(1.57–6.52)	3.19 (1.56–6.51)
	3 years ‐	134	3	405.27	7.40	1.11 (0.36–3.45)	0.73 (0.23–2.3)	0.74 (0.24–2.33)	0.78 (0.25–2.44)
MACE	No	19 562	1082	68 780.76	15.73	1(ref.)	1(ref.)	1(ref.)	1(ref.)
	‐ 1 year	72	25	180.66	138.38	8.33 (5.60–12.39)	4.56 (3.04–6.85)	4.64 (3.09–6.96)	4.71 (3.13–7.07)
	1–3 years	90	22	227.77	96.59	5.83 (3.82–8.89)	3.45 (2.25–5.3)	3.53 (2.30–5.44)	3.58 (2.33–5.51)
	3 years ‐	134	21	366.14	57.36	3.49 (2.26–5.37)	1.95 (1.26–3.03)	1.96 (1.26–3.05)	1.99 (1.28–3.10)

MACE: major adverse cardiac events including acute myocardial infarction, revascularization, and acute ischemic stroke.

Multivariate model 1: adjusted for age, sex, income, place of residence, hypertension, diabetes, dyslipidemia, COPD.

Multivariate model 2: adjusted for age, sex, income, place of residence, hypertension, diabetes, dyslipidemia, COPD, surgery type, radiotherapy, chemotherapy.

Multivariate model 3: (screening subgroup only): adjusted for age, sex, income, place of residence, hypertension, diabetes, dyslipidemia, COPD, surgery type, radiotherapy, chemotherapy, BMI, and smoking.

### Study outcomes according to follow‐up time since surgery

Within one year of follow‐up, prior PCI was associated with an increased risk of revascularization (HR = 2.35; 95% CI: 1.39–3.97), but not with death, ICU readmission, or stroke. However, with follow‐up for more than one year, the PCI group showed an increased risk of death (HR = 1.42; 95% CI: 1.19–1.70) and revascularization (HR = 5.15; 95% CI: 3.54–7.51) (Table 4, multivariate model 2). The Kaplan‐Meier survival curve is shown in Figure 2.

**Table 4:** Risk of study outcomes according to follow‐up time after surgery

	PCI before surgery	N	event	Duration	IR (per 1000)	Model 1	Model 2	Model 3
Within one year								
Death	No	30 237	1653	29 569.1	55.90	1(ref.)	1(ref.)	1(ref.)
	Yes	513	35	499.49	70.07	1.26 (0.90–1.76)	1.17 (0.83–1.65)	1.18 (0.84–1.67)
ICU readmission	No	30 237	1141	28 993.1	39.35	1(ref.)	1 (ref.)	1(ref.)
	Yes	513	29	487.43	59.50	1.51 (1.04–2.18)	1.04 (0.72–1.52)	1.05 (0.72–1.54)
Revascularization	No	30 237	121	29 503.32	4.10	1(ref.)	1(ref.)	1(ref.)
	Yes	513	17	491.83	34.56	8.42 (5.07–13.98)	2.33 (1.38–3.95)	2.35 (1.39–3.97)
Stroke	No	30 237	244	29 458.79	8.28	1(ref.)	1(ref.)	1(ref.)
	Yes	513	9	494.1	18.22	2.20 (1.13–4.28)	1.07 (0.54–2.12)	1.06 (0.54–2.10)
MACE	No	30 237	684	29 218.70	23.41	1(ref.)	1(ref.)	1(ref.)
	Yes	513	54	471.54	114.52	4.85 (3.68– 6.40)	2.11 (1.58– 2.81)	2.11 (1.58– 2.82)
After one year								
Death	No	28 593	6303	82 717.03	76.2	1(ref.)	1(ref.)	1(ref.)
	Yes	478	131	1161.02	112.83	1.45 (1.22–1.73)	1.31 (1.10–1.57)	1.42 (1.19–1.70)
ICU readmission	No	27 728	1516	78 488.96	19.31	1(ref.)	1(ref.)	1(ref.)
	Yes	453	26	1078.22	24.11	1.22 (0.82–1.79)	0.977 (0.66–1.45)	1.03 (0.70–1.54)
Revascularization	No	28 485	263	81 871.79	3.21	1(ref.)	1(ref.)	1(ref.)
	Yes	463	37	1067.42	34.66	10.92 (7.74–15.42)	5.14 (3.53–7.49)	5.15 (3.54–7.51)
Stroke	No	28 408	537	81 614.78	6.58	1(ref.)	1(ref.)	1(ref.)
	Yes	470	14	1118.88	12.51	1.90 (1.12–3.23)	1.45 (0.84–2.50)	1.52 (0.88–2.62)
MACE	No	19 562	1085	79 575.53	13.63	1(ref.)	1(ref.)	1(ref.)
	Yes	296	61	951.56	64.11	4.23 (3.27– 5.48)	2.66 (2.03– 3.48)	2.74 (2.09–3.60)

MACE, major adverse cardiac events including acute myocardial infarction, revascularization, and acute ischemic stroke.

Multivariate model 1: Crude.

Multivariate model 2: adjusted for age, sex, income, place of residence, hypertension, diabetes, dyslipidemia, COPD, surgery type, radiotherapy, chemotherapy.

Multivariate model 3: (screening subgroup only): adjusted for age, sex, income, place of residence, hypertension, diabetes, dyslipidemia, COPD, surgery type, radiotherapy, chemotherapy, BMI, and smoking.

## Discussion

To our knowledge, this is the first study to analyze the impact of prior PCI on long‐term outcomes in patients who underwent surgery for lung cancer, according to the time from PCI to surgery and follow‐up duration. As anticipated, we found that the PCI group had a higher risk than the non‐PCI group of death and revascularization after lung cancer surgery. Although our study did not reveal whether the cause of death was a cardiovascular event or cancer‐related, considering the higher prevalence of risk factors in the PCI group, we surmised that cardiovascular disease contributed to higher mortality. Moreover, acquired thrombophilia in lung cancer patients^25^ and perioperative chemo‐ and radiotherapy could have accelerated the progression of cardiovascular disease. To clarify, the thrombogenic effect of cisplatin‐based chemotherapy could play an essential role in the pathogenesis of myocardial infarction.^26^ Increased risk of restenosis after PCI and increased cardiac morbidity and mortality rates have been observed in patients treated with thoracic radiation.^27^

Our results are in line with those of previous studies. A USA study based on the Surveillance, Epidemiology, and End Results Program data showed similar results. Fernandez *et al*. demonstrated that the PCI group had inferior long‐term survival outcomes and were at higher risk for MACE, defined as death, myocardial infarction, stent thrombosis, and revascularization within 30 days after surgery.^6^ Kravchenko *et al*. analyzed the impact of cardiovascular comorbidity on the overall five‐year survival in NSCLC using cancer stage‐ and treatment‐specific groups. The results showed that comorbid myocardial infarction negatively affected survival in lung cancer patients who underwent surgery.^7^ However, these studies^6^, ^7^ did not conduct an analysis based on the time interval from PCI to surgery and the subsequent follow‐up duration.

Our study demonstrated that patients who underwent lung cancer surgery within one year after PCI were at the highest risk of revascularization and death, and risks were greatest for patients who received PCI within six months of surgery. Several studies have focused on how the time from PCI to surgery affected MACE after noncardiac surgery, although the results after lung cancer surgery were not analyzed separately. Consistent with our findings, these studies also demonstrated that the incidence of MACE was the highest when the interval between PCI and surgery was short (eg, within one year).^16^, ^17^, ^18^, ^19^, ^20^ However, these studies did not investigate whether patients who received PCI long before (eg, >1 year or > 3 years) were at higher risk of revascularization or death than the non‐PCI group, and some studies did not have a non‐PCI control group.^17^, ^19^, ^20^ Our study found an increased risk of death and revascularization in patients undergoing lung cancer surgery both within one year and one to three years after PCI. Therefore, clinicians should even pay special attention to patients who received PCI long before lung cancer surgery.

While most previous reports on the effect of prior PCI on postoperative outcomes presented only 30‐day or 90‐day outcomes,^16^, ^17^, ^18^, ^19^, ^20^ we analyzed the short‐ and long‐term survival results separately. Although the risk of revascularization was higher in the PCI group, there was no difference in mortality within one year of surgery according to PCI status, possibly because pulmonary complications or lung cancer itself were a common cause of death regardless of PCI status. However, mortality in the PCI group significantly increased after one year of follow‐up, indicating the increasing importance of assessing cardiovascular mortality in longer survivorship periods. In the same context, our data showed that the PCI group had higher risk of revascularization within one year of follow‐up, and even higher risk after one year.

The reasons for the significantly higher mortality and revascularization after one year of follow‐up need to be scrutinized thoroughly. Similar to our study, compared to the general population, patients who undergo PCI are known to have a higher rate of death, stroke, and MI over time,^28^ with stent‐related complications also increasing over time.^5^ Although the difference in risk was uncertain during the first year after surgery, the risk of death and revascularization were higher in the PCI group after one year from surgery. This could be explained by inappropriate long‐term management of lung cancer survivors. Smoking cessation is known to improve survival in NSCLC.^29^ However, many survivors resume smoking during the first year after lung cancer diagnosis.^30^ Although not investigated, smoking resumption could explain the findings, considering the higher prevalence of smokers in the PCI group. Furthermore, routine physical activity or exercise is recommended to decrease mortality in NSCLC survivors.^31^ However, patients who underwent lung cancer surgery showed reduced exercise capacity. Although most patients showed improvement within the first year,^32^ recovery in the PCI group in our study might have been delayed by the higher incidence of comorbidities.

Increased risk of ICU readmission was not evident in the PCI group. Previous studies showed that a pulmonary complication was the primary cause of ICU readmission after lung cancer surgery, with only approximately 10% cases attributable to cardiovascular complications (mostly cardiac arrhythmias).^21^ This suggested that complications related to coronary artery disease accounted for a small proportion of ICU readmissions. Therefore, our study may not have shown significant differences between the two groups due to the infrequency of complications associated with PCI.

Ischemic stroke and coronary artery disease share similar pathophysiology,^33^ and patients with a history of myocardial infarction or PCI have a higher risk of stroke during the follow‐up period.^34^ In this study, the risk of stroke was significantly greater only when PCI was performed within 1–3 years of surgery. It is hypothesized that during the first post‐operative year, the probability of stroke could decrease due to DAPT therapy after PCI. In addition, the number of occurrences of stroke were too small to accept these results as clinically meaningful findings.

We can draw clinical implications from this study. Among the patients who had a history of PCI, patients who survive one year after surgery may become confident and tend to neglect long‐term care. However, the risk of death increases one year after surgery, and patients require more careful cardiovascular risk management. Furthermore, careful management should begin within a year of receiving PCI treatment, and continue even if it has been more than three years.

There are several limitations to our study. First, as a retrospective analysis of administrative data, our study lacked detailed clinical information, such as cancer stage, pulmonary function test results, the cause of death (ie, cardiac or other), and the number of involved coronary arteries and degree of stenosis. Second, we did not determine whether patients received bare metal or drug‐eluting stents, which could have affected the results. Third, we did not have data on whether or when patients had stopped aspirin or DAPT during the perioperative period.

In conclusion, patients who underwent PCI before surgery for lung cancer were at both a short‐ and long‐term higher risk of revascularization. The risk was higher, particularly when the time interval between PCI and lung cancer surgery was less than one year. Moreover, the risk of revascularization and death remained higher than in the non‐PCI group at one year after surgery. More careful long‐term and perioperative management of cardiovascular risk is necessary for this population.

## Disclosure

The authors declare that there are no conflicts of interest.

## Supporting information


**Table S1** Risk of study outcomes according to interval between PCI and surgery
