# Supplementary material for: Impact of previous percutaneous coronary intervention on cardiovascular outcomes and mortality after lung cancer surgery: A nationwide study in Korea
Source: Thorac Cancer. 2020 Jul 12;11(9):2517–28. doi: 10.1111/1759-7714.13563 (PMC7471042; doi:10.1111/1759-7714.13563)
Supplement: Supplementary file 1 — Table S1 Risk of study outcomes according to interval between PCI and surgery [file TCA-11-2517-s001.docx]

**Supplementary Table 1**. Risk of study outcomes according to interval between PCI and surgery

| Outcomes | PCI before surgery | N | No. of events | person-years | rate (per 1,000 person-years) | Crude model | Multivariate model 1 | Multivariate model 2 | Multivariate model 3 |
| --- | --- | --- | --- | --- | --- | --- | --- | --- | --- |
| All patients |  |  |  |  |  |  |  |  |  |
| Death | No | 30,237 | 7,947 | 112,286.13 | 70.78 | 1(ref.) | 1(ref.) | 1(ref.) |  |
|  | - 6month | 84 | 34 | 256.98 | 132.3 | 1.86(1.33,2.61) | 1.72(1.22,2.41) | 1.87(1.33,2.63) |  |
|  | 6m - 1 year | 36 | 13 | 145.05 | 89.63 | 1.28(0.74,2.2) | 1.06(0.62,1.84) | 1.26(0.73,2.17) |  |
|  | 1-3 years | 152 | 51 | 513.44 | 99.33 | 1.40(1.06-1.84) | 1.30(0.99-1.72) | 1.45(1.09-1.91) |  |
|  | 3 years - | 241 | 68 | 745.04 | 91.27 | 1.28(1.01-1.63) | 1.16(0.91-1.47) | 1.17(0.92-1.49) |  |
| ICU  readmission | No | 30,237 | 2,655 | 107,482.06 | 24.7 | 1(ref.) | 1(ref.) | 1(ref.) |  |
|  | - 6month | 84 | 12 | 243.97 | 49.19 | 1.86(1.06,3.29) | 1.39(0.79,2.46) | 1.5(0.85,2.65) |  |
|  | 6m - 1 year | 36 | 5 | 132.08 | 37.86 | 1.56(0.65,3.74) | 1.11(0.46,2.68) | 1.21(0.5,2.92) |  |
|  | 1-3 years | 152 | 15 | 495.52 | 30.27 | 1.19(0.72-1.98) | 0.93(0.56-1.55) | 0.97(0.58-1.62) |  |
|  | 3 years - | 241 | 23 | 694.08 | 33.14 | 1.25(0.83-1.89) | 0.93(0.62-1.41) | 0.94(0.62-1.42) |  |
| Revascularization | No | 30,237 | 384 | 111,375.11 | 3.45 | 1(ref.) | 1(ref.) | 1(ref.) |  |
|  | - 6month | 84 | 18 | 221.07 | 81.42 | 23.31(14.51,37.42) | 8.5(5.22,13.84) | 8.72(5.35,14.21) |  |
|  | 6m - 1 year | 36 | 5 | 136.46 | 36.64 | 10.71(4.43,25.88) | 3.73(1.53,9.11) | 3.73(1.53,9.11) |  |
|  | 1-3 years | 152 | 9 | 492.95 | 18.26 | 5.28(2.73-10.22) | 2.19(1.12-4.28) | 2.18(1.11-4.25) |  |
|  | 3 years - | 241 | 21 | 708.77 | 29.63 | 8.57(5.52-13.30) | 3.26(2.07-5.13) | 3.21(2.04-5.05) |  |
| Stroke | No | 30,237 | 781 | 111,073.57 | 7.03 | 1(ref.) | 1(ref.) | 1(ref.) |  |
|  | - 6month | 84 | 3 | 246.36 | 12.18 | 1.71(0.55,5.3) | 1.12(0.36,3.49) | 1.15(0.37,3.58) |  |
|  | 6m - 1 year | 36 | 0 | 145.05 | 0 | - | - | - |  |
|  | 1-3 years | 152 | 9 | 487.75 | 18.45 | 2.6(1.35-5.02) | 1.69(0.87-3.29) | 1.78(0.92-3.47) |  |
|  | 3 years - | 241 | 11 | 733.82 | 14.99 | 2.10(1.16-3.81) | 1.35(0.74-2.46) | 1.36(0.75-2.50) |  |
| MACE | No | 30,237 | 1,869 | 108,794.22 | 17.18 | 1(ref.) | 1(ref.) | 1(ref.) |  |
|  | -6m | 84 | 24 | 202.11 | 118.75 | 6.43(4.3, 9.62) | 3.45(2.3, 5.2) | 3.54(2.35, 5.32) |  |
|  | 6m - 1 year | 36 | 11 | 127.05 | 86.58 | 5.08(2.81,9.18) | 2.58(1.42, 4.68) | 2.7(1.48, 4.9) |  |
|  | 1-3 years | 152 | 35 | 419.4 | 83.45 | 4.66(3.33, 6.51) | 2.55(1.82,3.58) | 2.66(1.89, 3.73) |  |
|  | 3 years - | 241 | 45 | 674.53 | 66.71 | 3.71(2.76, 4.99) | 2.01(1.49, 2.72) | 2.01(1.49, 2.72) |  |
| Screening subgroup |  |  |  |  |  |  |  |  |  |
| Death | No | 19,562 | 4,670 | 70,839.56 | 65.92 | 1(ref.) | 1(ref.) | 1(ref.) | 1(ref.) |
|  | - 6month | 53 | 18 | 150.21 | 119.84 | 1.81(1.14,2.88) | 1.72(1.08,2.74) | 1.84(1.15,2.93) | 1.88(1.18,3.00) |
|  | 6m - 1 year | 19 | 7 | 70.47 | 99.33 | 1.52(0.63,3.32) | 1.19(0.57,2.5) | 1.47(0.7,3.08) | 1.43(0.68,3) |
|  | 1-3 years | 90 | 33 | 293.79 | 112.33 | 1.71(1.21-2.40) | 1.62(1.15-2.29) | 1.75(1.24-2.47) | 1.75(1.24-2.48) |
|  | 3 years - | 134 | 38 | 405.4 | 93.73 | 1.43(1.04-1.97) | 1.29(0.94-1.78) | 1.32(0.95-1.82) | 1.30(0.94-1.79) |
| ICU readmission | No | 19,562 | 1,597 | 67,911.73 | 23.52 | 1(ref.) | 1(ref.) | 1(ref.) | 1(ref.) |
|  | - 6month | 53 | 4 | 145.24 | 27.54 | 1.07(0.4,2.85) | 0.81(0.3,2.16) | 0.83(0.31,2.23) | 0.84(0.31,2.24) |
|  | 6m - 1 year | 19 | 3 | 67.31 | 44.57 | 1.93(0.62,6.00) | 1.37(0.44,4.28) | 1.51(0.48,4.7) | 1.48(0.47,4.6) |
|  | 1-3 years | 90 | 8 | 283.82 | 28.19 | 1.17(0.59-2.35) | 0.94(0.47-1.89) | 0.97(0.48-1.95) | 0.97(0.48-1.96) |
|  | 3 years - | 134 | 13 | 375.2 | 34.65 | 1.38(0.80-2.39) | 1.05(0.60-1.82) | 1.05(0.61-1.83) | 1.04(0.60-1.80) |
| Revascularization | No | 19,562 | 219 | 70,323.90 | 3.11 | 1(ref.) | 1(ref.) | 1(ref.) | 1(ref.) |
|  | - 6month | 53 | 14 | 127.39 | 109.9 | 35(20.34,60.22) | 13.23(7.52,23.28) | 13.52(7.68,23.81) | 14.15(8.02,24.95) |
|  | 6m - 1 year | 19 | 4 | 63.04 | 63.45 | 20.41(7.59,54.89) | 6.87(2.51,18.78) | 6.88(2.51,18.88) | 6.95(2.52,19.11) |
|  | 1-3 years | 90 | 7 | 279.48 | 25.05 | 8.06(3.80-17.10) | 3.52(1.63-7.57) | 3.57(1.66-7.70) | 3.61(1.68-7.79) |
|  | 3 years - | 134 | 9 | 383.98 | 23.44 | 7.51(3.85-14.63) | 2.99(1.51-5.92) | 2.92(1.47-5.80) | 2.87(1.44-5.71) |
| Stroke | No | 19,562 | 464 | 70,100.28 | 6.62 | 1(ref.) | 1(ref.) | 1(ref.) | 1(ref.) |
|  | - 6month | 53 | 1 | 144.62 | 6.91 | 1.04(0.15,7.42) | 0.75(0.1,5.35) | 0.74(0.1,5.24) | 0.76(0.11,5.44) |
|  | - 1 year | 19 | 0 | 70.47 | 0 | - | - | - | - |
|  | 1-3 years | 90 | 8 | 269.7 | 29.66 | 4.47(2.22-8.99) | 3.14(1.54-6.40) | 3.20(1.57-6.52) | 3.19(1.56-6.51) |
|  | 3 years - | 134 | 3 | 405.27 | 7.4 | 1.11(0.36-3.45) | 0.73(0.23-2.3) | 0.74(0.24-2.33) | 0.78(0.25-2.44) |
| MACE | No | 19,562 | 1,082 | 68,780.76 | 15.73 | 1(ref.) | 1(ref.) | 1(ref.) | 1(ref.) |
|  | -6m | 53 | 19 | 119.66 | 158.78 | 9.34(5.93, 14.71) | 5.45(3.43, 8.65) | 5.4(3.4, 8.57) | 5.68(3.57, 9.03) |
|  | 6m - 1 year | 19 | 6 | 61 | 98.36 | 6.21(2.78, 13.85) | 3.01(1.34, 6.76) | 3.21(1.43, 7.2) | 3.05(1.36, 6.85) |
|  | 1-3 years | 90 | 22 | 227.77 | 96.59 | 5.83(3.82, 8.89) | 3.45(2.25, 5.3) | 3.53(2.30, 5.44) | 3.58(2.33, 5.51) |
|  | 3 years - | 134 | 21 | 366.14 | 57.36 | 3.49(2.26, 5.37) | 1.95(1.26, 3.03) | 1.96(1.26, 3.05) | 1.99(1.28, 3.10) |

MACE: major adverse cardiac events including acute myocardial infarction, revascularization, and acute ischemic stroke

Multivariate model 1: adjusted for age, sex, income, place of residence, hypertension, diabetes, dyslipidemia, COPD
Multivariate model 2: adjusted for age, sex, income, place of residence, hypertension, diabetes, dyslipidemia, COPD, surgery type, radiotherapy, chemotherapy

Multivariate model 3 (Screening subgroup only): adjusted for age, sex, income, place of residence, hypertension, diabetes, dyslipidemia, COPD, surgery type, radiotherapy, chemotherapy, BMI, and smoking
